# Posterior Reversible Leukoencephalopathy With Hemorrhagic Features: A Case Series

**DOI:** 10.7759/cureus.49587

**Published:** 2023-11-28

**Authors:** Franco Appiani, Carlos Santiago Claverie, Francisco R Klein

**Affiliations:** 1 Neurology, Favaloro Foundation University Hospital, Buenos Aires, ARG; 2 Diagnostic Unit, Barcelona Alzheimer Treatment and Research Center (ACE), Barcelona, ESP; 3 Intensive Care Unit, Favaloro Foundation University Hospital, Buenos Aires, ARG

**Keywords:** brain injury and seizure, neurocritical care unit, reversible posterior cerebral edema syndrome, magnetic resonance imaging, transplantation, cerebral hemorrhage

## Abstract

Introduction

Posterior reversible leukoencephalopathy syndrome (PRES) is a clinical-radiological condition characterized by reversible subcortical vasogenic cerebral edema of acute or subacute onset in circumstances that disrupt capillary permeability, unfrequently accompanied by cytotoxic and/or hemorrhagic lesions. We describe a case series of PRES with hemorrhagic features.

Subjects and methods

Electronic medical records of hospitalized patients diagnosed with PRES from January 2009 to December 2021 were collected. Demographic data, medical history, clinical presentation, and outcome were recorded. Variables were compared between patients with and without hemorrhagic features using the Wilcoxon-Mann-Whitney test with a statistical significance level of p<0.05.

Results

Over a 12-year period, 33 patients were diagnosed with PRES, of whom 10 had hemorrhagic features: seven cortical microbleeds, two intraparenchymal hematomas, and one subarachnoid hemorrhage. Half of the patients were women, with a median age of 45.8 years (interquartile range (IQR) 21.8), and were admitted for non-neurological reasons. The sample included nine transplant recipients (six solid organa, three bone marrowa), with four patients in the immediate post-transplant period. PRES occurred in the context of infections and blood pressure fluctuations under cytotoxic drugs, such as immunosuppressants. Seventy percent showed improvement/resolution on neuroimaging at a median of 70 days (IQR 62.9). The three major hemorrhages occurred in the context of thrombocytopenia. The recorded in-hospital mortality was 10%. When compared to PRES without hemorrhagic features, patients with hemorrhagic features had a lower use of corticosteroids (50% vs. 78.8%; p=0.02) and a higher presence of restrictive lesions on neuroimaging (60% vs. 17%; p=0.04), with no differences in the other analyzed variables.

Conclusion

Patients with PRES and hemorrhagic features had a lower use of corticosteroids and a higher presence of restrictive lesions on neuroimaging. Further studies are needed to better understand the clinical implications and management of PRES with hemorrhagic manifestations.

## Introduction

Posterior reversible leukoencephalopathy syndrome (PRES) is a clinical-radiological condition characterized by reversible subcortical vasogenic cerebral edema of acute or subacute onset. It manifests with a diverse array of symptoms, encompassing headache, seizures, altered consciousness, visual disturbances, and other focal neurological signs. [1] It has received different denominations, although the most widely used term remains PRES [2].

The hallmark of this disorder is impaired cerebral autoregulation, leading to dysfunction of the blood-brain barrier. The triggering mechanism is still not fully understood and may not be unique. Both cerebral hyperperfusion and endothelial injury can increase vascular permeability, predominantly in posterior areas with less sympathetic innervation, resulting in characteristic vasogenic edema. Therefore, PRES typically occurs in specific clinical settings such as blood pressure fluctuations, use of cytotoxic drugs, and preeclampsia/eclampsia, among others [3].

The diagnosis is based on clinical suspicion associated with specific imaging findings. However, atypical features such as cytotoxic edema or hemorrhage are frequently observed in PRES [4,5]. While some prior studies have highlighted the presence of hemorrhagic manifestations in a subgroup of PRES patients, reports detailing this specific presentation remain scarce. The association of these findings with clinical presentation and prognosis lacks clarity in the existing literature, thereby motivating our study. This study aims to describe a case series of patients diagnosed with PRES and presenting hemorrhagic features.

## Materials and methods

This observational study utilized electronic medical records of hospitalized patients diagnosed with PRES from January 2009 to December 2021. The reviewed data comprised demographic information, medical history, clinical presentation, imaging findings, time to improvement or resolution on imaging, and in-hospital mortality. Imaging studies were conducted using a nuclear magnetic resonance machine (1.5 T, Philips® Achieva; Philips, Eindhoven, Netherlands) and a computed tomography scanner (Toshiba® Aquilion Prime; Toshiba, Tokyo Japan).

PRES with hemorrhagic features was defined by the presence of deoxyhemoglobin or methemoglobin detected on gradient echo or susceptibility-weighted imaging sequences of magnetic resonance imaging (MRI), or spontaneous hyperdensity observed in cranial computed tomography (CT) by prespecified neuroimaging criteria. Based on their distribution and size, these features were categorized as intraparenchymal hematoma, subarachnoid hemorrhage, or microhemorrhage (less than 5 mm)/superficial siderosis. Improvement or resolution was determined by a minimum 50% reduction in cerebral edema observed in subsequent imaging during follow-up. The evolution of these images was analyzed, covering all scans conducted after diagnosis until the conclusion of follow-up or death.

Systemic infection was defined as a clinical presentation or microbiological findings requiring antibiotic or antiviral treatment. Blood pressure fluctuations were evaluated based on daily metrics and clinical criteria. A positive evaluation was determined by a 24-hour variation of 30 mmHg compared to the baseline or if vasopressor drugs were necessary. Blood analysis noted abnormal values in hematology, coagulation studies, and relevant ions, such as hemoglobin < 12 g/dL and magnesium < 1 mg/dL, among others. Medications administered up to five days before diagnosis were categorized into antimicrobials, vasopressors/inotropes, red blood cell infusions, chemotherapeutic agents, and immunosuppressants. Recorded therapeutic initiatives included anti-seizure drugs and pharmacological measures for blood pressure control.

Variables were compared based on the presence of hemorrhagic features using the Wilcoxon-Mann-Whitney test, with a predetermined level of statistical significance set at p < 0.05. Statistical analysis was conducted using R (version 2022; R Development Core Team, Vienna, Austria). Informed consent was obtained from all individual participants included in the study, and the study protocol received approval from the Favaloro Foundation University Hospital Ethics Committee.

## Results

Over 12 years (2009-2021), 33 patients with clinical and neuroimaging findings compatible with PRES were collected. Among them, 10 had hemorrhagic features: seven had microhemorrhages, two had intraparenchymal hematomas, and one had subarachnoid hemorrhage.

In describing the group with hemorrhagic features (10), half of the patients were female (five) with a median age of 45.8 years (interquartile range (IQR) 21.8). The group consisted of transplant recipients (nine) and non-transplant recipients (one). The diagnosis occurred in non-neurological hospital admissions (nine), such as systemic infections or scheduled surgeries, with a median hospital stay of 57.4 days (IQR 49.48).

Among transplant recipients with PRES and hemorrhagic features, six patients had solid organ transplants (two cardiac, two hepatic, one intestinal, and one pulmonary), and three had bone marrow transplants. In four patients, PRES occurred within two weeks of transplantation (bone marrow, lung, heart, and liver). Relevant clinical histories included three patients with hypertension and two with chronic kidney disease.

Among the in-hospital complications in those with PRES and hemorrhagic features, notable findings included systemic infections (eight), blood pressure fluctuations (six), vasopressor requirements (five), anemia (three) with red blood cell transfusion criteria (two), and severe hypomagnesemia requiring intravenous supplementation (three). At the time of PRES, they were receiving immunosuppressive drugs such as tacrolimus (eight), mycophenolate (three), rapamycin (two), cyclosporine (two), or chemotherapy agents such as busulfan and etoposide (two). Corticosteroid therapy was observed in half of the cases at diagnosis (five), as shown in Table 1.

**Table 1:** Demographic characteristics, personal history, and immunosuppression reported in patients with severe leukoencephalopathy, with and without hemorrhagic features 1. SD: standard deviation 2. Reason for hospitalization due to neurological symptoms compatible with clinical presentation of PRES (posterior reversible leukoencephalopathy syndrome) 3. MRI: magnetic resonance imaging 4. Improvement of at least 50% of initial vasogenic edema in brain magnetic resonance imaging 5. CKD: Chronic kidney disease 6. It includes different chemotherapeutic agents with different mechanisms of action

	Total		Hemorrhagic features				
Demographics	n	%/SD^ 1^	NO	%/SD	YES	%/SD	p-value
Total number (n)	33	(100)	23	(77)	10	(33)	
Female (%)	20	(61)	15	(65)	5	(50)	NS
Age	44.15	(20.1)	43.43	(19.9)	45.80	(21.8)	NS
Hospitalization due to PRES (%)^2^	6	(18.2)	5	(21.7)	1	(10)	NS
Days to presentation and improvement							
Total hospitalization days	44.00	(38.7)	38.17	(32.6)	57.40	(49.4)	NS
Days of hospitalization until PRES in brain MRI^3^	16.85	(21.5)	14.65	(17.9)	21.90	(28.6)	NS
Days from PRES MRI to MRI control	87.85	(184.7)	111.32	(212.5)	24.14	(17.1)	NS
Days from PRES MRI to MRI improvement^4^	122.08	(205.5)	139.61	(233.9)	69.50	(62.9)	NS
Improvement in follow-up MRI^4^	25	(75.7)	18	(78.3)	7	(70)	NS
In-hospital mortality	11	(33.3)	10	(43.5)	1	(10)	NS
Personal history (%)							
Background of CKD^5^	15	(45)	13	(56)	2	(20)	NS
History of arterial hypertension	13	(40)	10	(43)	3	(30)	NS
Last transplant (%)							
Bone marrow	5	(15)	2	(9)	3	(30)	NS
Solid organ	24	(73)	18	(78)	6	(60)	NS
No transplant	4	(12)	3	(13)	1	(10)	NS
Immunosuppression at PRES (%)							
Tacrolimus	20	(61)	12	(52)	8	(80)	NS
Mycophenolate	12	(36)	9	(39)	3	(30)	NS
Cyclosporine	9	(27)	7	(30)	2	(20)	NS
Azathioprine	3	(9)	3	(13)	0	(0)	NS
Rapamycin	3	(9)	1	(4)	2	(20)	NS
Corticosteroids	26	(79)	21	(91)	5	(50)	0.02
Use of chemotherapy^6^	11	(33)	9	(39)	2	(20)	NS

In the only non-transplant recipient with hemorrhagic features, PRES occurred on postoperative day 21 after coronary revascularization surgery in the context of arterial hypertension and post-surgical systemic infection. Neuroimaging revealed microhemorrhages both supra- and infratentorial.

The most frequent combination of symptoms that raised suspicion of PRES in this group of patients with hemorrhagic features did not differ from the group without these features. In all cases, at least one initial brain MRI was performed, as detailed in Table 2.

**Table 2:** Clinical presentation and neuroimaging features in patients with cerebral leukoencephalopathy, with and without hemorrhagic features 1. SD: standard deviation 2. PRES: Posterior reversible leukoencephalopathy 3. Clinical or microbiological infection requiring antibiotic or antiviral treatment 4. Evaluated with daily metrics and according to clinical criteria 5. Less than 12 g/dL in women and 13g/dL in men 6. According to clinical criteria, associated or not with decreased blood values 7. Serum magnesium concentration < 1.8 mg/dL (< 0.70 mmol/L)

	Total		Hemorrhagic features				
Clinical presentation of PRES (%)^2^	n	%/SD^1^	NO	%/SD	YES	%/SD	p-value
Headache	10	(30)	9	(39)	1	(10)	NS
Visual disturbance	16	(48)	11	(48)	5	(50)	NS
Altered consciousness	11	(33)	8	(35)	3	(30)	NS
Seizure	19	(58)	15	(65)	4	(40)	NS
Nonconvulsive status epilepticus	3	(10)	2	(9)	1	(10)	NS
Seizure status epilepticus	2	(6)	1	(5)	1	(10)	NS
Distribution of cerebral edema (%)							
Bilateral	28	(85)	18	(78)	10	(100)	NS
Unilateral	3	(9)	3	(13)	0	(0)	NS
Frontal	16	(48)	13	(56)	3	(30)	NS
Temporary	11	(33)	8	(35)	3	(30)	NS
Parietal	22	(65)	15	(65)	7	(70)	NS
Occipital	21	(64)	15	(65)	6	(60)	NS
Basal ganglia	5	(15)	3	(13)	2	(20)	NS
Brainstem	4	(12)	4	(17)	0	(0.0)	NS
Cerebellum	4	(12)	3	(13)	1	(10)	NS
Cortical	4	(12)	3	(13)	1	(10)	NS
Subcortical	6	(18)	6	(26)	0	(0)	NS
Cortico-subcortical	19	(58)	11	(48)	8	(80)	NS
Restriction	10	(30)	4	(17)	6	(60)	0.04
Disorders concomitant with PRES							
Systemic infections^3^	20	(61)	12	(52)	8	(80)	NS
Fluctuations in blood pressure^4^	19	(58)	13	(56)	6	(60)	NS
Requirement of vasopressors	14	(42)	9	(39)	5	(50)	NS
Anemia^5^	13	(39)	10	(43)	3	(30)	NS
Red blood cell requirement^6^	14	(42)	12	(52)	2	(20)	NS
Severe hypomagnesemia^7^	8	(24)	5	(22)	3	(30)	NS

**Figure 1:**
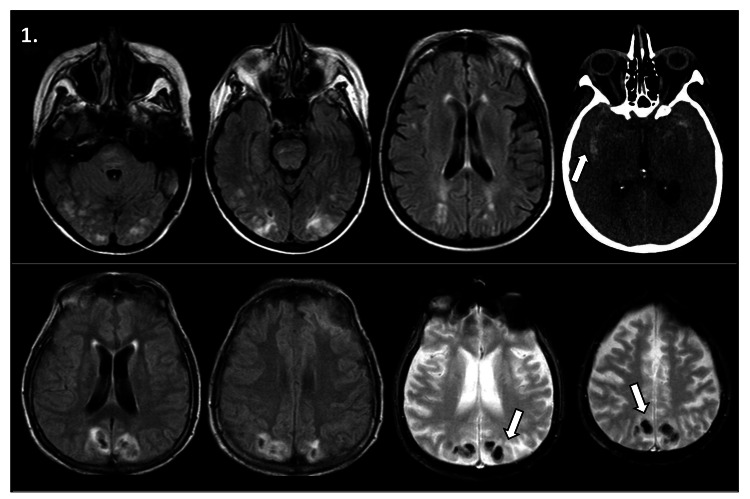
Hemorrhages in the context of reversible posterior leukoencephalopathy syndrome Two different patients, one in the upper panel and another in the lower panel (Upper panel, center and left images) Axial brain magnetic resonance imaging (MRI) in FLAIR sequence, showing bioccipital cortico-subcortical and brainstem hyperintense areas consistent with vasogenic edema. (Upper panel, far right image) Axial brain computed tomography (CT) 48 hours later, demonstrating hyperdense areas in both Sylvian fissures compatible with subarachnoid hemorrhage. (Lower panel, center and left images) Brain MRI in FLAIR sequence displaying bioccipital cortico-subcortical hyperintense areas. (Lower panel, center and right images) Brain MRI in GRE sequence exhibiting concentric hypodense areas in the bioccipital region consistent with intraparenchymal hematoma.

The only subarachnoid hemorrhage recorded in this series occurred 48 hours after PRES in a bone marrow transplant recipient who died during hospitalization, as evidenced in Figure 1. The two recorded intraparenchymal hematomas were located in one patient at the right basal frontal level and in another patient at a biparietal level surrounded by perilesional vasogenic edema. The neuroimages depicting the latter can be seen in Figure 1. Among the three patients encountering significant bleeding (two intracerebral hematomas and one subarachnoid hemorrhage), all experienced thrombocytopenia post-transplantation.

**Figure 2:**
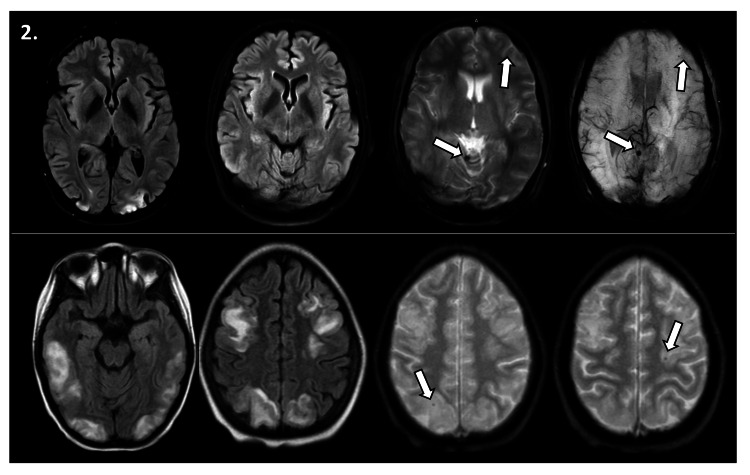
Microhemorrhages in the context of reversible posterior leukoencephalopathy syndrome Two different patients, one in the upper panel and another in the lower panel. (Upper panel, center, and left images) Axial brain magnetic resonance imaging (MRI) in FLAIR sequence, showing bioccipital hyperintense areas consistent with vasogenic edema. (Upper panel, center, and right images) GRE and SWI sequences, respectively, demonstrating small hypointense areas indicative of microhemorrhages. These microhemorrhages are observed in the infratentorial region of the cerebellar vermis and in the supratentorial left frontal lobe. (Lower panel, center, and left images) Extensive bilateral frontal, occipital, and temporal vasogenic edema areas. (Lower panel, center, and right images) Isolated microhemorrhages in the same regions as the described vasogenic edema.

Microhemorrhages emerged as the predominant imaging feature within our sample of patients displaying hemorrhagic characteristics. These microhemorrhages were notably observed both distantly from the edematous regions and within the same affected areas, as depicted in Figure 2.

In our subset of PRES patients displaying hemorrhagic features, a follow-up MRI was conducted with a median delay of 24.1 days (IQR 17.1). Among these, seven patients (70%) exhibited improvement or resolution, with a median duration of 69.5 days (IQR 62.9). However, follow-up imaging was not available for two patients due to loss of follow-up, and one patient displayed worsening in the subsequent image and deceased during hospitalization.

Within the PRES group with hemorrhagic features, two patients encountered epileptic status, one with convulsive seizures and the other with non-convulsive seizures. Cerebrospinal fluid analysis was conducted in five patients, with two showing albumin-cytologic dissociation, and one having a significant number of red blood cells associated with intraparenchymal hematoma.

Blood pressure management was carefully monitored in all cases, and eight patients received antiseizure medications. No new deaths occurred post-discharge, but two out of the 10 cases were lost to follow-up. The in-hospital mortality rate for this group stood at 10%.

When comparing the analyzed variables between patients with PRES and hemorrhagic features and those without these features (n=23), no significant differences were found. Notably, lower utilization of corticosteroids was observed in those with hemorrhagic manifestations (50% vs 78.8%; p=0.02), as detailed in Table 1. Furthermore, a higher incidence of diffusion-restricted lesions was noted (60% vs 17%; p=0.04), as demonstrated in Table 2.

## Discussion

We have presented a series comprising 10 patients diagnosed with PRES, exhibiting hemorrhagic features on neuroimaging. Most of these individuals underwent transplantation and received treatment with immunosuppressive and/or cytotoxic therapies. Among all the registered PRES patients during the study, approximately 30.3% displayed hemorrhagic characteristics. In our assessment of clinical presentation and therapeutic approaches, we noted minimal differences between patients with and without hemorrhagic manifestations, except for a lower use of corticosteroids in the hemorrhagic group. Notably, the relationship between corticosteroid treatment and hemorrhagic manifestations in PRES remains unreported in existing literature.

Hemorrhage in PRES appears multifactorial, potentially involving hemodynamic disturbances, endothelial injury, coagulopathy arising from clinical conditions or therapies, and underlying comorbidities, with significant emphasis on endothelial/blood-brain barrier breakdown. Its manifestation includes microhemorrhages, focal hematomas, and subarachnoid hemorrhages. The available evidence regarding this particular presentation in PRES is limited, and its association with established pro-coagulation factors, such as anticoagulant therapies or thrombocytopenia, lacks consistency [1,6-8]. In a case series featuring PRES with intracerebral hemorrhage, a notable prevalence of bleeding diathesis was observed, and only a minority of patients exhibited favorable clinical outcomes [7]. However, other authors conclude that abnormalities in the coagulation profile do not appear to be a prerequisite for the occurrence of hemorrhage in PRES because of its small prevalence in positive cases from a large case series [6,9]. In our study, the occurrence of three cases exhibiting hematomas or subarachnoid hemorrhages coincided with post-transplant thrombocytopenia. The only in-hospital deceased registered evolved 24 hours after PRES with a fatal subarachnoid hemorrhage. The analysis of platelet count, anticoagulant use, or coagulopathies as differential factors between the two groups could not be performed due to the retrospective nature of the study, which relied on medical records lacking such data.

Existing evidence suggests varying reversibility in PRES, where microhemorrhages, even without other major bleedings, may indicate greater endothelial dysfunction and an unfavorable prognosis [8-11]. Our series predominantly featured microhemorrhages, showing a tendency towards less resolution in follow-up neuroimaging, although not statistically significant. Conversely, the presence of restricted diffusion shows contradictory and scarce results in the literature [12]. In our study, MRI diffusion-restricted lesions were noted mainly in PRES with hemorrhagic features. Despite an association between hemorrhagic features and a higher presence of restricted lesions, no increased mortality was observed in our study.

Evidence of PRES with hemorrhagic features in transplant patients is scarce. Limited case reports or series, including transplant patients, observed this complication [6,7,13-15]. Numerous variables, including organ transplantation, immunosuppression therapy, hemodynamic parameters, coagulation profile, blood pressure, and other comorbidities, are likely to play a role in the occurrence of hemorrhage in this setting. Although our case series lack statistical significance, a trend toward a higher prevalence of hemorrhagic feature, mainly in bone marrow transplant, was observed.

The retrospective and single-center design, along with the small sample size, absence of hierarchical variable analysis (such as anticoagulation or thrombocytopenia), and limited evaluation beyond in-hospital mortality, are notable limitations of our study. These factors emphasize the imperative for larger, more comprehensive investigations. Subsequent investigations should independently assess the effects of microhemorrhages and major bleeding on patient outcomes. Additionally, there should be dedicated analyses considering the status of transplant patients, aiming to refine the knowledge and management of these infrequently PRES presentations in distinct populations.

## Conclusions

In summary, our study examined 10 patients with PRES displaying hemorrhagic features on neuroimaging, constituting approximately 30.3% of our PRES cases. While no significant clinical or therapeutic differences were observed between patients with and without hemorrhagic manifestations, we did note a lower use of corticosteroids in the hemorrhagic group. Moreover, hemorrhagic features were associated with more restricted lesions on neuroimaging, but not increased mortality.

Further research is essential to better comprehend the clinical implications and management of PRES with hemorrhagic features, with a focus on differentiating between microhemorrhages and major bleedings to improve care for these uncommon presentations.
